# Enzymatic insights into an inherited genetic disorder

**DOI:** 10.7554/eLife.31127

**Published:** 2017-09-14

**Authors:** Liping Zhang, Kelly G Ten Hagen

**Affiliations:** National Institute of Dental and Craniofacial ResearchNational Institutes of HealthBethesdaUnited States

**Keywords:** NGLY1, BMP signaling, midgut, *D. melanogaster*

## Abstract

Mutations in an enzyme involved in protein degradation affect a signaling pathway that stimulates the development of the digestive tract.

**Related research article** Galeone A, Han SY, Huang C, Hosomi A, Suzuki T, Jafar-Nejad H. 2017. Tissue-specific regulation of BMP signaling by Drosophila N-glycanase 1. *eLife* **6**:e27612. doi: 10.7554/eLife.27612

DNA sequencing has been very successful in identifying mutations associated with human genetic disorders, but understanding how the disruption of particular genes results in disease remains a constant challenge. One such example of this is the identification of mutations in a gene known as *NGLY1* in patients with an inherited disorder (Need et al., 2012). *NGLY1* encodes an enzyme that is found in the cytoplasm of all cells and is responsible for removing sugar chains known as N-glycans from proteins that are destined to be degraded (Figure 1A; Suzuki et al., 1993; Suzuki et al., 2016). Patients harboring mutations in this gene present with a multitude of symptoms, including delayed development, peripheral nerve disorders and low muscle tone (Enns et al., 2014; Caglayan et al., 2015; Lam et al., 2017). However, it is not clear how the loss of this enzyme leads to these symptoms.

**Figure 1.**
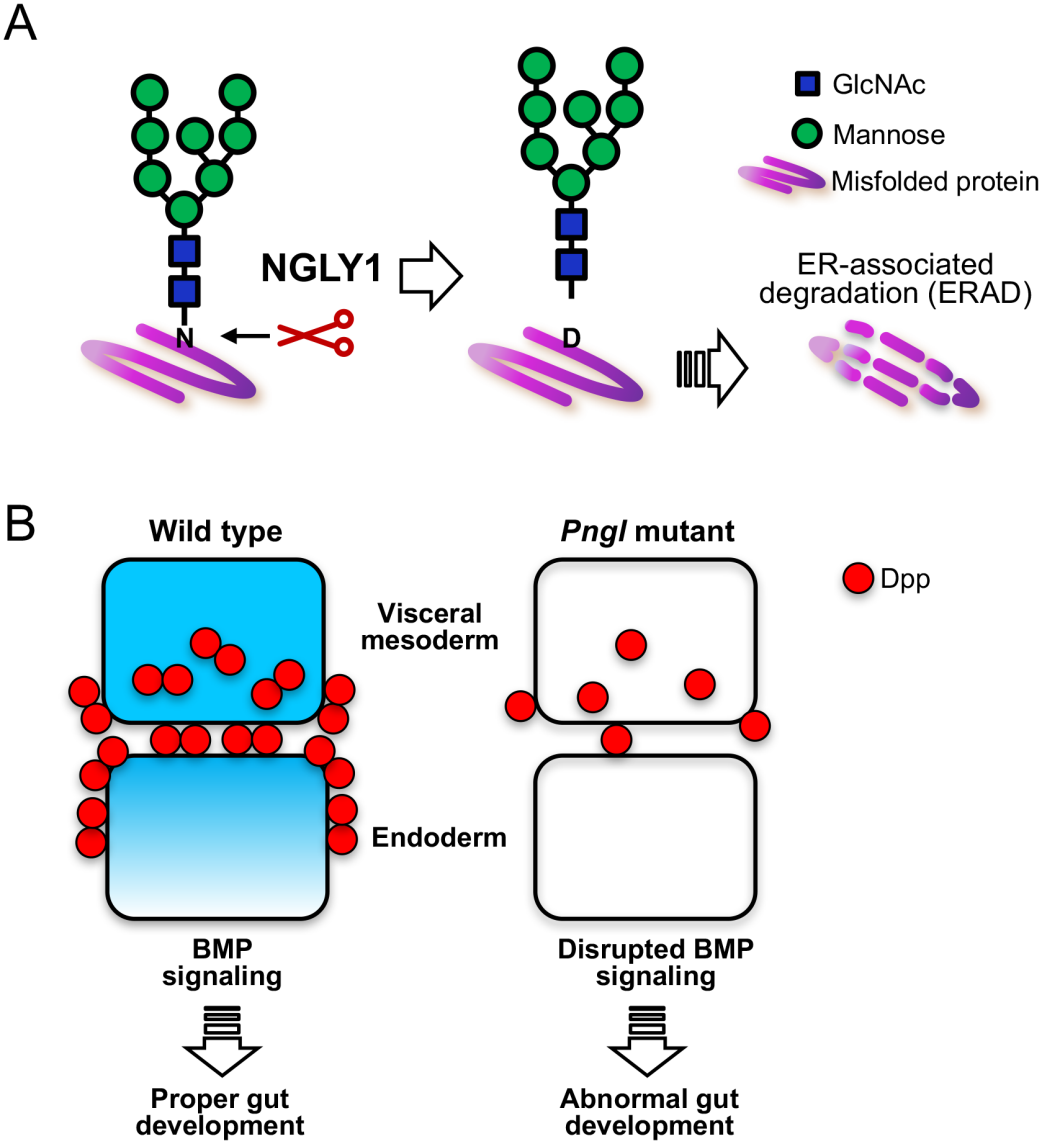
The role of NGLY1 in protein processing and development. (**A**) In humans and other animals the NGLY1 enzyme removes sugar chains called N-glycans (shown in green and blue) from proteins (purple) that are destined to be degraded via a process known as endoplasmic reticulum-associated degradation (ERAD). If NGLY1 removes a sugar chain from the amino acid asparagine (N), the latter becomes a different amino acid, aspartic acid (D), which may alter the activity of the protein. (**B**) The equivalent of the NGLY1 enzyme in the fruit fly is known as Pngl. In fruit flies, BMP signaling stimulates the development of the digestive system (left). Homodimers of a BMP ligand called Dpp (red) in a layer of tissue known as the visceral mesoderm (upper layer) activate BMP signaling, which then signals to cells in the endoderm (lower layer). Dpp fails to properly form homodimers in fruit flies with mutations in the *Pngl* gene, which disrupts BMP signaling within both the visceral mesoderm and the endoderm (right). Cells with active BMP signaling are shown in blue. GlcNAc, *N*-acetylglucosamine.

Now, in eLife, Hamad Jafar-Nejad and colleagues at Baylor College of Medicine and the RIKEN Global Research Cluster – including Antonio Galeone as first author – report new insights into the role of this enzyme in the development of the fruit fly, *Drosophila melanogaster* (Galeone et al., 2017). Fruit flies have served as excellent models for studying many aspects of human development and disease over the years. Taking advantage of sophisticated genetic tools unique to the fly, Galeone et al. show that the loss of a gene called *Pngl* – which is the fruit fly equivalent of *NGLY1* – causes portions of the digestive tract to be malformed. Further experiments trace these defects back to when the digestive tract begins to form in the embryo and reveal that a signaling pathway known as bone morphogenetic protein (or BMP) signaling is disrupted in cells that will give rise to portions of the digestive tract.

The BMP pathway is of particular interest because it regulates many aspects of development in both fruit flies and mammals (Wang et al., 2014). Cells secrete molecules known as BMP ligands that then bind to specific receptors on the surface of cells to stimulate BMP signaling cascades (O'Connor et al., 2006). BMP ligands exist as dimers containing either two identical ligand molecules (homodimers) or two different ones (heterodimers). Galeone et al. demonstrate that a BMP ligand called Dpp forms fewer homodimers in the developing gut of flies with mutations in *Pngl*. This leads to defects in BMP signaling in two layers of tissue that are required for the digestive system to form properly (Figure 1B).

This study is the first to identify a specific signaling pathway that is disrupted in the absence of the Pngl enzyme and may provide insight into the underlying causes of some of the symptoms typically seen in patients with mutations in the *NGLY1* gene. Whether patients have similar alterations in BMP signaling within specific cells or tissues remains to be determined, but this study will help to inform future investigations.

The findings of Galeone et al. raise a number of questions regarding how this enzyme works. An enzymatically inactive version of Pngl did not rescue specific digestive system defects, suggesting that Pngl activity is important for BMP signaling. However, it is not known whether the enzyme acts directly on Dpp to affect its ability to form homodimers. It is also possible that the loss of Pngl could affect BMP signaling indirectly, by influencing how other proteins within these cells are modified or degraded. BMP signaling within the affected cells may be uniquely sensitive to disruptions in the normal systems that regulate proteins at this stage of development. Additionally, not all phenotypes within the *Pngl* mutants are due to defects in BMP signaling, suggesting that the Pngl enzyme also affects other proteins and pathways. Direct examination of the N-glycans attached to Dpp and other proteins in wild type and *Pngl*-deficient tissues will begin to address some of these questions.

Finally, studies on the role of NGLY1 in other organisms suggest that this enzyme may have roles beyond the removal of N-glycans on proteins destined for degradation (Dalet et al., 2011; Figure 1A). There is also evidence suggesting NGLY1 has a role in cells that is independent of its enzymatic activity (Maerz et al., 2010). Regardless, the study by Galeone et al. demonstrates that loss of *Pngl* results in tissue- and stage-specific alterations in the BMP signaling pathway and provides a new lens through which we can begin to dissect how it influences other systems.
